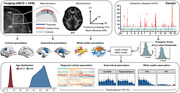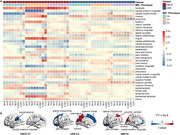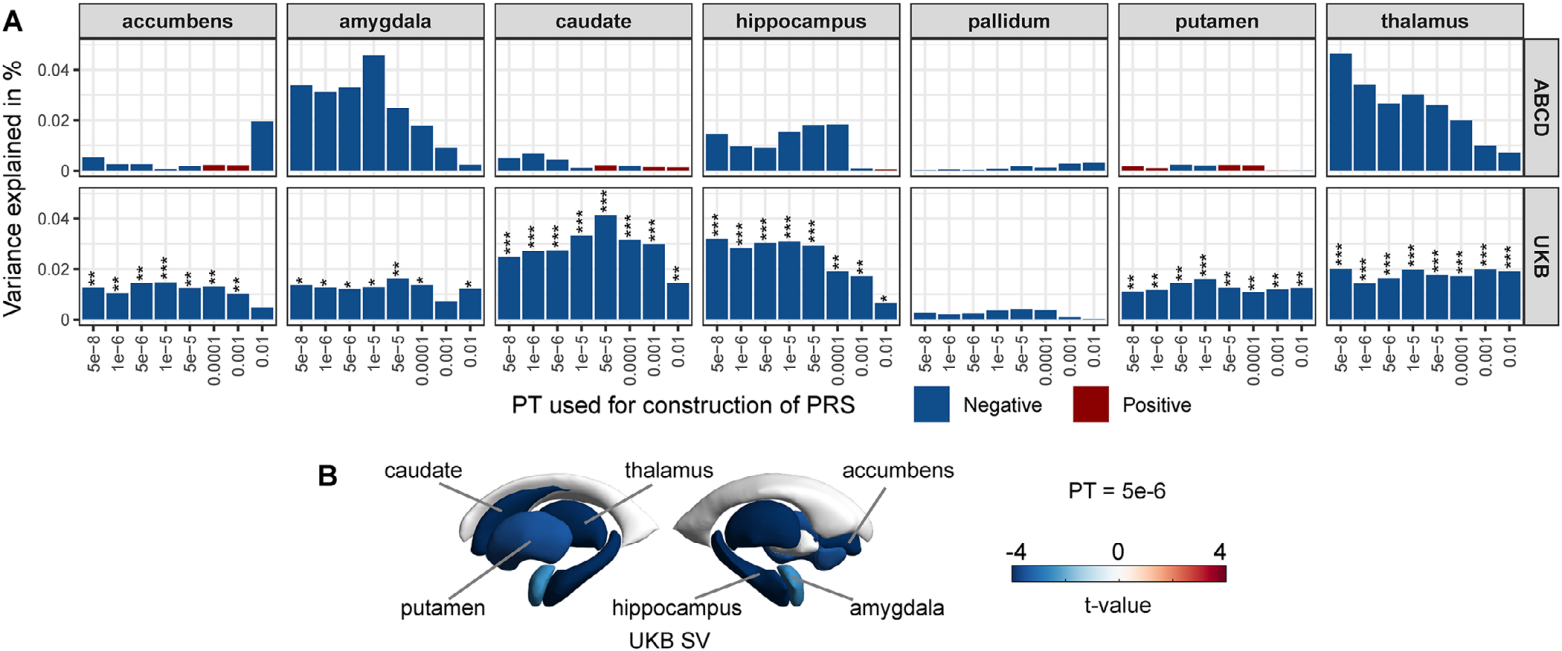# Association between polygenic risk for Alzheimer’s disease and brain structure in children and adults

**DOI:** 10.1002/alz.087408

**Published:** 2025-01-09

**Authors:** Xiao‐Yu He, Bang‐Sheng Wu, Jin‐Tai Yu

**Affiliations:** ^1^ Huashan Hospital, Fudan University, Shanghai China; ^2^ National Center for Neurological Disorders, Shanghai China

## Abstract

**Background:**

Alzheimer’s disease (AD), a complex and polygenic disease with a considerable hereditary component (60–80%), is a progressive neurodegenerative disorder characterized by concealed onset, and individuals often have significant cognitive impairment and histopathological changes in the brain before overt clinical diagnosis. However, the correlations between genetic risk for Alzheimer’s disease (AD) with comprehensive brain regions at a regional scale are still not well understood. We aim to explore whether these associations vary across different age stages.

**Method:**

This study used large existing genome‐wide association datasets to calculate polygenic risk score (PRS) for AD in two populations from the UK Biobank (N ∼ 23 000) and Adolescent Brain Cognitive Development Study (N ∼ 4660) who had multimodal macrostructural and microstructural magnetic resonance imaging (MRI) metrics. We used linear mixed‐effect models to assess the strength of the association between AD PRS and multiple MRI metrics of regional brain structures at different stages of life.

**Result:**

Compared to those with lower PRSs, adolescents with higher PRSs had thinner cortex in the caudal anterior cingulate and supramarginal. In the middle‐aged and elderly population, AD PRS had correlations with regional structure shrink primarily located in the cingulate, prefrontal cortex, hippocampus, thalamus, amygdala, and striatum, whereas the brain expansion was concentrated near the occipital lobe. Furthermore, both adults and adolescents with higher PRSs exhibited widespread white matter microstructural changes, indicated by decreased fractional anisotropy (FA) or increased mean diffusivity (MD).

**Conclusion:**

In conclusion, our results suggest genetic loading for AD may influence brain structures in a highly dynamic manner, with dramatically different patterns at different ages. This age‐specific change is consistent with the classical pattern of brain impairment observed in AD patients.